# Healthy Eating Habits among the Population of Serbia: Gender and Age Differences

**Published:** 2015-03

**Authors:** Ana Đ. Jovičić

**Affiliations:** Geographical Institute “Jovan Cvijić” of the Serbian Academy of Sciences and Arts, Belgrade, Serbia

**Keywords:** Diet habits, Healthy eating, Population, Serbia

## Abstract

The purpose of the study is to examine healthy eating habits of the population of Serbia through three dimensions: knowledge, problems, and feelings as well as to determine whether there are any differences between genders and among different age-groups. The research instrument was an Eating Habits Questionnaire (EHQ) which consisted of 35 items. There were 382 respondents involved in the study. The reliability and factor structure of the questionnaire were verified by using factor analysis. The results of MANOVA showed that there is a significant difference in the habits concerning healthy eating between men and women [F (3,378)=4.26, p=0.006; Wilks’ Lambda=0.97]. When the results for the dependent variables (knowledge, problems, and feelings) were considered separately, it was determined that there is no significant difference between men and women, which confirms the results of the *t*-test. The effect of age on the three dimensions of healthy eating habits was examined within three age-groups, by using ANOVA. The results showed that knowledge about healthy eating increases with age [F (2,379)=6.14, p=0.002] as well as positive feelings which occur as a result of healthy eating [F (2,379)=3.66, p=0.027]. Unlike ANOVA, MANOVA showed difference among the age-groups only when it came to the ‘knowledge’ variable. This study is important as it shows the current state of awareness on healthy eating habits in the researched populace and may be the basis for further research in this field in Serbia.

## INTRODUCTION

In the scientific community, it is generally agreed that diet is the most important factor in maintaining health. Healthy eating is considered to be one of the most important means of promoting health (1). Healthy eating contributes to an overall sense of wellbeing (2) and is a cornerstone in the prevention of many common chronic diseases (3). Healthy and unhealthy eating are both influenced by a variety of individual and collective (social and environmental) factors (4).

Healthy eating represents a balanced and varied diet, consisting of healthy foods: fresh and natural foods, plenty of fruits and vegetables, and foods containing vitamins and minerals (5). It also includes eating habits and behaviours that are consistent with improving, maintaining, and/or enhancing health—both physical and psychological (4).

The notion of healthy eating can often be confusing and unclear to individuals (6), regardless of the fact that nutrition knowledge is widespread today. Various studies show that the respondents often relate healthy foods to certain types of food, such as fruits and vegetables (5,7,8), food with low caloric value (9,10), notions, such as ‘balanced’ diet or eating ‘proper meals’ (11), and fresh groceries (12). A qualitative study (13) has shown that the complex and diverse explanations of the study participants concerning healthy eating reflected their personal, social and cultural experiences as well as their environments.

Various studies analyze psychological variables which affect the behaviour relating to healthy eating (14–16). Hayes (17) found that health concern and appearance are the most significant motivational factors in applying healthy eating. According to numerous studies, consumers are increasingly interested in their own health, which is possible to improve by changing their eating habits (18,19).

This is the first study in Serbia, which analyzes the habits of the population concerning healthy eating. This study is important as it shows the current state of awareness regarding healthy eating in Serbia, and it may help in formulating a strategy which would be the basis for promoting healthy eating. This study analyzes healthy eating habits of the population of Serbia through three dimensions: knowledge, problems, and feelings. ‘Knowledge’ is related to which extent people think they apply the principles of healthy eating. ‘Problems’ is concerned with difficulties related to healthy eating. ‘Feelings’ is related to positive emotions which individuals have for healthy eating.

The objective of this study is to determine whether there are any differences between genders and among different age-groups within the dimensions of knowledge, problems, and feelings.

## MATERIALS AND METHODS

The pilot study was conducted in the Republic of Serbia between March 2011 and January 2012. An online and paper form of the questionnaire in Serbian language was used for the study. The paper form of the questionnaire was distributed in 550 copies, and 323 were returned to the author (response rate 58%). The study used only those sets of questionnaire that were filled out completely. There was a total of 276 fully-completed questionnaire. An additional 106 sets of questionnaire were acquired online. There were no statistically significant differences [t (380)=-0.14, p=0.989] between the respondents who participated in the paper-based survey (1.93±0.51) and the online survey (1.93±0.52). The data collection was done through random sampling. Participation in this study was voluntary and anonymous.

The paper form of the questionnaire was distributed in elementary schools, high schools, university, and retirement homes. To conduct the study, approval was required from the principals and teachers. The students filled in the questionnaire during the break between classes. In the university, students filled in the questionnaire during the lecture sessions, with their professors’ permission. In retirement homes, the author distributed the questionnaire to the owners, which were then further distributed to the residents.

Google Docs (Google Inc, USA), which is a freeware web-based office suite was used in creating the online questionnaire. The settings were such that no respondent could return an incomplete questionnaire. The online questionnaire was posted on the Facebook Fan Page of a large Serbian retail chain, which has units across the country. Also, the author distributed the online questionnaire to friends, acquaintances, and colleagues via email, asking them to fill it out but also forward it to anyone they thought would be interested in contributing to the study.

The questionnaire consisted of two parts. The first part was related to sociodemographic variables while the second part consisted of the Eating Habits Questionnaire (EHQ) with 35 items (20). The respondents were expected to state the level of their agreement with the statements on a 4-point Likert scale (1=incorrect, 2=partially correct, 3=mostly correct, and 4=correct).

The EHQ instrument was originally created by Graham (20) to assess cognitions, behaviours, and feelings relating to pathological fixation on healthy eating. Within the two pilot studies, Graham developed the instrument and examined its psychometric characteristics. The factor structured of 35 items was examined in the first study, and 3 factors were kept: knowledge, problems, and feelings. In the first study, the internal validity of the questionnaire was confirmed with high internal consistency coefficients for the subscales (0.87 to 0.91). Within the second study, a confirmatory factor analysis was conducted, and 21 items were kept. The study samples for both studies were based on the feedback from students, which is why the original studies cannot be generalized.

The study presented in this paper is a pilot study, and it is the first stage of a larger analytical investigation about the eating habits of the population of Serbia. To determine the factors and the structure of the items, which are adjusted to the conditions in Serbia, Graham's 35-item version of the questionnaire was used.

### Data analysis

The data were processed by applying the statistical SPSS package (version 19.0) (IBM Corporation, USA). The data analysis has covered frequency analysis, descriptive analysis, factor analysis, the Independent-samples *t*-test, the One-way Analysis of Variance (ANOVA), and the Multivariate Analysis of Variance (MANOVA). The *t*-test, ANOVA, and MANOVA are statistical models used in analyzing differences between group means. The *t*-test analyzes differences between two group means while ANOVA deals with more than two groups. Statistically significant differences occur if the p value is less than 0.05. Categorical independent variable, which can be a continuous variable reduced to a small number of categories and a continuous dependent variable, are necessary for conducting the abovementioned tests. MANOVA analyzes differences between groups on linear combinations of multiple quantitative variables. MANOVA uses one categorical independent variable and two or more continuous, dependent variables.

Before using ANOVA and MANOVA, the normality of the distribution scores for the dependent variables was performed. The Kolmogorov-Smirnov test showed non-significant result (p>0.05) but the actual shape of normal probability plots suggested a reasonably straight line, which indicated a normal distribution of dependent variables.

Applying research methodology developed by a certain culture with respondents of a different cultural ambient can lead to misinterpretation of the acquired data, which is why the factor structure and the internal consistency of the respondents were examined. The EHQ instrument has never been used with the population of Serbia before, which is why the exploratory factor analysis was used. Exploratory factor analysis is used in order to identify the number and the nature of the latent variables, determine the structure through summarizing the data, and to reduce the data with minimal data loss. The Cronbach's alpha coefficient, which is an average correlation between the items on the scale, was used as an estimate of the reliability of the instrument. Its value is between 0 and 1 where a higher value shows higher reliability. Nunnally (21) gives a recommended alpha value of at least 0.70.

Before conducting factor analysis, the suitability of the data for factor analysis was assessed. By reviewing the correlation matrix, many coefficients of value 0.30 and higher were discovered. The value of the Kaiser-Meyer-Olkin Criterion was 0.91, which exceeds the recommended value of 0.60 (22). The Bartlett's test of sphericity (23) has reached the statistical significance (p<0.001) which points out to the factorability of the correlation matrix.

## RESULTS

The following sociodemographic characteristics of the respondents were analyzed: gender, age, occupation, education, place of residence, monthly income, and whether the respondents share their place of residence with anyone (Table 1). The average age was 25.63±13.56 years, with the youngest respondent being 11 years old, and the oldest being 78 years old. The average age of men was 26.36±15.31 years while the average age of women was 25.17±12.34 years.

By analyzing the main components, the presence of six values over one was discovered, which explains for 27.9%, 38.2%, 44.0%, 48.1%, 51.5%, and 54.9% of the variance. By reviewing the scree plot, the existence of a clear breaking point above the fourth component was discovered. Based on the Cattell Scree Test (24), it was decided to keep the three components as it was the case with the original questionnaire.

**Table 1.** Sociodemographic variables of the respondents

Demographical characteristics	Number (%) 382 (100%)
Gender	
Male	147 (38.5)
Female	235 (61.5)
Age (years)	
Below 21	165 (43.2)
21-30	138 (36.1)
Over 30	79 (20.7)
Occupation	
Student	264 (69.1)
Employed	88 (23.0)
Unemployed	16 (4.2)
Retired	14 (3.7)
Years of education	
4-8	137 (35.9)
9-12	103 (27.0)
13-16	102 (26.7)
Over 17	40 (10.4)
Place of residence	
Urban	296 (77.5)
Rural	86 (22.5)
Monthly income (Euro)	
No income	232 (60.7)
Below 200	27 (7.1)
201-400	47 (12.3)
401-600	53 (13.9)
Over 601	23 (6.0)
Do you live with anyone?	
Alone	38 (9.9)
Friends	21 (5.5)
Family	257 (67.3)
Partner	66 (17.3)

The three-factor solution explained a total of 44.0% of the variance where the contribution of the first component was 27.9%, of the second one was 10.3%, and of the third one was 5.8%. To interpret the components easier, an Oblimin rotation was conducted. The rotated solution showed that the components had seven or more weight factors. To reduce and improve the scale, only the weight factors over 0.5 were kept, which is 23 out of a total of 35 items. The interpretation of the three components was in compliance with the previous research (20). There is a small correlation between the factors, which are reported in the Component Correlation Matrix within the results of the factor analysis: r=0.340 between factors one and two, r=-0.411 between factor one and three, and r=-0.236 between factor two and three.

The results of this pilot study demonstrate that when examining the habits of the Serbian population concerning healthy eating, it is necessary to analyze the following factors:

Factor 1: Knowledge—8 items,
Factor 2: Problems—8 items,
Factor 3: Feelings—7 items.

**Table 2.** Descriptive statistics and factor loadings

Modified EHQ Item number and name	Number (%) 382 (100%)	
	Mean±SD	Factor loadings
Knowledge	Chronbach's alpha=0.865	
1. My diet is healthier than most diets	2.17±0.93	0.727
2. I place more and more restrictions on the foods I can eat	2.55±1.01	0.608
3. I turn down social offers that involve eating unhealthy food	2.14±1.09	0.570
10. I follow a health-food diet rigidly	1.75±0.92	0.676
11. My diet is better than other people's diets	2.18±0.96	0.720
18. My eating habits are superior to others’	1.72±0.89	0.625
27. I eat only healthy foods	1.85±0.95	0.733
32. I prepare food in the healthiest way	1.94±0.95	0.624
Average factor scores (EFA)	2.04±0.69	
Problems	Chronbach's alpha=0.838	
14. I spend more than three hours a day thinking about healthy food	1.31±0.72	0.582
17. I think about healthy food when engaged in other activities	1.52±0.81	0.700
24. I avoid going out to eat with others because of my diet	1.20±0.58	0.734
28. Most of my free time revolves around healthy eating	1.36±0.71	0.672
29. In the past year, friends or family members have told me that I'm overly concerned with healthy eating	1.26±0.64	0.585
31. I am distracted by thoughts of healthy eating	1.25±0.59	0.624
34. I go out less since I began healthy eating	1.34±0.74	0.678
21. I daydream about eating healthy	1.61±0.96	0.522
Average factor scores (EFA)	1.36±0.50	
Feelings	Chronbach's alpha=0.865	
4. I feel peaceful when I eat healthy	2.94±1.07	-0.717
6. Healthy eating brings me fulfillment	2.58±1.06	-0.726
7. I have made efforts to eat healthy over time	2.96±0.98	-0.604
12. I feel in control when I eat healthy	2.39±1.08	-0.695
22. I feel great when I eat healthy	2.54±1.07	-0.741
26. I love healthy eating	2.77±1.07	-0.730
33. It's important to me to eat healthy	2.88±1.02	-0.537
Average factor scores (EFA)	2.72±0.78	

Table 2 shows the results of the component factor analysis as well as a proposal on how to modify the questionnaire to examine the healthy eating habits of the population of Serbia. There are a total of 23 items in the modified questionnaire.

The internal consistency of the modified questionnaire measured with the Chronbach's alpha is 0.904 for the entire questionnaire, which exceeds the recommended value of 0.7 (21). The result inside the three subscales is as follows: the ‘knowledge’ factor is 0.865, the ‘problems’ factor is 0.838, and the ‘feelings’ factor is 0.865.

Pearson's correlation coefficient shows statistically significant and positive correlations between three factors. The ‘knowledge’ and the ‘problems’ factor correlation is r=0.358, p<0.001; the ‘knowledge’ and the ‘feelings’ factor correlation is r=0.623, p<0.001, and the ‘problems’ and the ‘feelings’ factor correlation is r=0.317, p<0.001.

Table 2 also shows that the mean ranks and the standard deviation of the kept items were the highest values within the ‘feelings’ factor, such as: “I have made efforts to eat healthy over time” (2.96), “I feel peaceful when I eat healthy” (2.94), “It is important to me that I eat healthy” (2.88); the lowest mean ranks within the ‘problems’ factor: “I am distracted by thoughts of eating healthy” (1.25) and “My friends and family told me that I am overly concerned with eating healthy” (1.26).

A one-way between-groups multivariate analysis of variance (MANOVA) was performed to investigate gender differences on healthy eating habits on the combination of dependent variables: ‘knowledge’, ‘problems’, and ‘feelings’. Preliminary assumption testing was conducted to check for normality, linearity, homogeneity of variance-covariance matrices, and multicollinearity, with no serious violations noted. There was a statistically significant difference between men and women on the combined dependent variables: [F (3,378)=4.26, p=0.006; Wilks’ Lambda=0.97]. When results for the dependent variables were considered separately, it was determined that there is no significant difference between men and women.

To confirm the results of the previous analysis, an additional analysis was conducted. Table 3 shows the mean ranks for men (knowledge 2.09±0.67; problems 1.38±0.54; feelings 2.63±0.81) and women (knowledge 2.00±0.69; problems 1.34±0.46; feelings 2.78±0.75). By using the *t*-test, the research results of ‘knowledge’, ‘problems’, and ‘feelings’ factor concerning healthy eating were compared, and it was determined that there is no significant difference between men and women (p>0.05) (Table 3).

By using the One-way Analysis of Variance (ANOVA), the effect of age on the ‘knowledge’, ‘problems’, and ‘feelings’ concerning healthy eating for the three age-groups was examined (Group 1: under 21; Group 2: 21-30, and Group 3: over 30).

A statistically significant difference was determined for the ‘knowledge’ factor on a p<0.05 level, with the results in three groups [F (2,379)=6.14, p=0.002]. The Eta squared=0.03 points out low difference according to the Cohen's criteria (25). Additional comparison using the Tukey's HSD test points out to the difference between the first (1.96±0.65) and the second group (1.99±0.66) compared to the third group (2.28±0.69). No significant difference was determined between the groups (Group 1: 1.35±0.51; Group 2: 1.38±0.51; Group 3: 1.34±0.48) under the ‘problems’ factor [F (2,379)=0.26, considering p>0.05]. The ‘feelings’ factor concerning healthy eating was perceived differently [F (2,379)=3.65 p=0.027] between the groups. A difference between Group 1 (2.63±0.75) and Group 3 (2.92±0.77) was determined by using an additional test and the Eta squared=0.01, which means that the actual statistical difference according to the Cohen's criteria is small. There were no statistical differences between the second age-group (2.72±0.80) when compared with the first and the third age-group.

The additional MANOVA test was performed to investigate age differences (Group 1: under 21; Group 2: 21-30, and Group 3: over 30) regarding the healthy eating habits. There was a statistically significant difference between age-groups on the combined dependent variables [F (6,754)=3.57, p=0.010; Wilks’ Lambda=0.96; partial Eta squared=0.02]. When the results for the dependent variables were considered separately, the only difference to reach statistical significance, using a Bonferroni-adjusted alpha level of 0.017, was for ‘knowledge’ [F (2,379)=6.14, p=0.002, partial Eta squared=0.03]. An inspection of the mean scores indicated that the third group is different compared to the first and the second group, which is already presented in ANOVA. Thus, the results of MANOVA are different from the results of ANOVA. Considering the three age-groups, a statistical significance of the dependent variables was found only in the case of the ‘knowledge’ variable while, in ANOVA, this was the case with variables ‘knowledge’ and ‘feelings’.

**Table 3.** Results of the Independent-samples *t*-test according to the gender variable

Variables	Men (N=147) Mean±SD	Women (N=235) Mean±SD	*t*-test	
			t value	p value
Knowledge	2.09±0.68	2.00±0.69	1.177	0.240
Problems	1.38±0.54	1.34±0.46	0.730	0.466
Feelings	2.63±0.81	2.78±0.75	-1.811	0.071

Degree of freedom (Df)=380

SD=Standard deviation

p=Level of significance

## DISCUSSION

The results of the study conducted on a sample of 382 respondents showed that there is a significant difference between men and women in the habits concerning healthy eating. However, if the variables that analyze healthy eating habits (knowledge, problems, and feelings) are examined separately, there are no gender differences. The study showed that knowledge about healthy eating increases with age as well as the positive feelings which occur as a result of healthy eating also increase.

To interpret the study results properly, further explanation on the ‘knowledge’ factor is necessary. The ‘knowledge’ factor in this context includes the application of healthy eating principles and the behaviour which is related to the selection of healthy food, its preparation and consumption. This factor covers declarative and procedural knowledge about healthy eating (26). Apart from motivation, knowledge is a factor which significantly affects healthy eating. However, having knowledge about a certain thing does not always mean direct application. When a person is motivated, the knowledge of dietary recommendations may affect his/her behaviour. The perceived benefits of healthy eating may also affect behaviour only if a person feels that healthy eating is relevant for him/her, if he/she is motivated and has sufficient knowledge to change his/her behaviour (27). Knowledge necessary for the application of healthy eating principles also involves nutritional knowledge. Various studies have shown that better nutritional knowledge is related to better eating habits (28–30). Also, Goode *et al.* (31) found that increased knowledge of a healthy diet is one of the most important reasons in changing a diet. In this sense, the educational and health organizations should inform and influence the consumption of healthy foods and the acquisition of a healthy lifestyle. The study results showed that knowledge about healthy eating increases with age. Older population, aged over 31 years, is more concerned with the selection of healthy food, its preparation and consumption, which is not the case with the younger population. Some studies point out that the “culture of healthy eating” should start from an early childhood (32–34). Campaigns for improving the public health, formal education as well as all the other information that individuals acquire by different media, such as Internet forums and blogs, contribute to nutrition knowledge to a high extent (35). This can further affect individual behaviour and concern about healthy eating.

There is a connection between social context and the amount of food consumed. People tend to eat more when they are in the company of people who eat more and less when their companion eats less (36). Healthy eating may, sometimes, become the reason for decreased social contacts. However, this was not the case in this study, considering the mean ranks of the items: “I go out less since I began eating healthy” (1.34±0.74), or “I avoid going out to eat with others because of my diet.” (1.20±0.58). The results have shown that the respondents do not perceive healthy eating in a negative context (1.36±0.50), which shows that they do not find healthy food to affect social interactions negatively and that it does not lead to the psychological disorders relating to healthy eating. For example, modern trends and the new lifestyle impose healthy food as a choice but also a burden which, in certain cases, can become an obsession. The disorder of extreme application of the principles of healthy eating is called Orthorexia Nervosa. Bratman describes orthorexia as a contemporary phenomenon which is characterized by the “obsession of healthy and proper nutrition” (37–41).

Feelings and emotions are related to eating in two ways. First, emotions may affect an individual's preferences and eating behaviour (42,43). For example, negative emotions, such as sadness, anger, and fear can increase impulsive food consumption. Positive emotions, such as pleasure, can affect the increase of food pleasantness and consumption of healthy foods (44–47). Second, eating certain types of food and trying different tastes may induce positive or negative feelings in an individual. For example, the study shows that there is a relation between the consumption of chocolate and the feelings of happiness and guilt. Happiness is an emotion induced by enjoying the taste of chocolate while guilt appears as an effect of consumption of chocolate which can negatively affect the slenderness and weight in women (48). The results showed that healthy eating induces positive feelings in an individual (2.72±0.78) and that they increase with years. ANOVA has determined that positive emotions which occur as a result of healthy eating are more pronounced with respondents who are over 30 years compared to respondents below 21 years of age. The study of Croll *et al.* showed that, in general, healthy eating was not important to adolescents (49). This may explain lower average values on the ‘feelings’ scale in the age-group below 21 years. Another study conducted on students showed that healthy eating is helpful in providing positive feelings (50). As people age, they tend to care more about healthy eating since its benefits will be more apparent and noticeable (51). The study results might be explained by the fact that positive emotions can occur as a result of the feeling of control the individual has in preventing disease, maintaining optimal weight and achieving attractive appearance (17).

Data presented by the Institute of Public Health of Serbia (52) point out certain problems relating to the population's diet, which indicates that healthy eating is still not a dominant choice amongst the population of Serbia. Serbia is a country stricken by political and economical problems and has been going through a difficult transition period for years. A healthy eating trend has been prevalent in Serbia in the recent years. Considering the large number of residents who are in an unfavourable socioeconomic position, the price of healthy foods (53,54) may be one of the reasons why it does not have prevalence among the population of Serbia. Still, through public institutions, non-profit organizations and primarily educational institutions, it is necessary to strive for the education of the population and to raise awareness about the significance of healthy eating by various campaigns. These beneficial efforts could result in the improvement of the overall health of the Serbian population in the future.

### Limitations

Although this is a unique study related to healthy eating in Serbia, there are several limitations. The sample is relatively small, with a low response rate. Most of the respondents are students (elementary school, high school, and university). Also, by using the online questionnaire, an arbitrary population of respondents is covered. All of the above indicate that there is no evidence of the possibility to generalize these results to different genders or different age-groups as well as the population of Serbia in general. The interpretation should be taken with a certain level of reservation. In addition, the instrument used eliminates the possibility of comparison with other study results. The factor structure of the questionnaire and its reliability should be examined on a larger sample in Serbia, and the study should include the respondents from different strata.

### Conclusions

The instrument used in this study needs further development and testing, and this can be the basis for future research on healthy eating habits. Further research should include other demographic variables as well as other social and environmental factors when analyzing eating habits.

## ACKNOWLEDGEMENTS

The paper is the result of research within a project [III 47007] funded by the Ministry of Education, Science and Technological Development of the Republic of Serbia.

**Conflict of interest:** Authors declare no competing interests.
